# Unseen Aggressor: A Case of Amelanotic Melanoma With Orbital Metastasis and Imaging-Pathology Correlation

**DOI:** 10.7759/cureus.88333

**Published:** 2025-07-19

**Authors:** Azalea Guadalupe Altamirano De La Cruz, César Alberto Santoyo Reza, Francisco Javier Lugo Rincón-Gallardo, Abraham Ramírez Saavedra, Karen Alejandra Diaz Mendoza

**Affiliations:** 1 Internal Medicine, Mexican Social Security Institute (IMSS) General Zone Hospital No. 8, Ensenada, MEX; 2 Internal Medicine, Mexican Social Security Institute (IMSS) General Zone Hospital No. 57, Cuautitlán Izcalli, MEX; 3 Internal Medicine, General Hospital of the Institute of Social Security and Services for State Workers, Querétaro, MEX; 4 Internal Medicine, Hospital Angeles Metropolitano, Mexico City, MEX; 5 General Medicine, Autonomous University of Baja California, Tijuana, MEX

**Keywords:** amelanotic malignant melanoma, cutaneous dermal metastasis, dermoscopy findings, late recurrence, skeletal metastases

## Abstract

Amelanotic melanoma represents an uncommon and aggressive variant of cutaneous melanoma. The characteristic absence of pigmentation poses significant diagnostic challenges, frequently leading to delayed clinical recognition and unfavorable prognosis compared to conventional pigmented melanomas.

We report a 57-year-old Caucasian male patient with a medical history of amelanotic melanoma of the right foot surgically treated in 2010. The patient subsequently lost medical follow-up and presented 13 years later with nonspecific left upper quadrant abdominal pain. Comprehensive evaluation revealed extensive metastatic disease involving multiple organ systems. Chest computed tomography demonstrated bilateral pulmonary nodules with characteristic "cannonball" morphology, while brain magnetic resonance imaging revealed multiple intraparenchymal lesions with surrounding vasogenic edema. Physical examination disclosed multiple erythematous cutaneous nodules on the thorax and extremities, bilateral lymphadenopathy, and hepatosplenomegaly.

Dermoscopic examination of cutaneous lesions revealed pathognomonic "milky-red areas" and polymorphous atypical vascular patterns, including irregular linear, dotted, and corkscrew vessels. Histopathological analysis of a cervical lymph node biopsy confirmed metastatic epithelioid amelanotic melanoma, with immunohistochemistry demonstrating positive staining for S100, Melan-A, and CDX10, while cytokeratin AE1/AE3 was negative.

This case exemplifies the unpredictable biology of amelanotic melanoma, an uncommon and aggressive variant of cutaneous melanoma. Underscoring the critical importance of lifelong dermatological surveillance, advanced imaging techniques, and dermoscopic evaluation in managing this aggressive malignancy. Contemporary immunotherapy and targeted therapies offer significant therapeutic advances for patients with advanced metastatic disease, emphasizing the need for multidisciplinary management and sustained clinical vigilance in optimizing patient outcomes.

## Introduction

Amelanotic melanoma represents a rare and diagnostically challenging variant of cutaneous melanoma, comprising 2-8% of cases [1,2]. Characterized by absent pigmentation, this entity frequently evades timely recognition, contributing to advanced stages at diagnosis and poorer prognoses than conventional melanomas [1,3]. Clinically, it manifests as erythematous nodules, plaques, or depigmented papules that mimic benign lesions (e.g., pyogenic granuloma, Spitz nevus, seborrheic keratosis) or other malignancies (e.g., basal cell carcinoma, cutaneous lymphoma) [1,3]. Diagnostic difficulties persist due to this clinical similarity and the lesion's depigmented appearance [4,5].

This challenge is exacerbated by its predilection for Fitzpatrick type I Caucasians >50 years, who often present with asymptomatic nodules [1,2,6]. Consequently, initial management must prioritize precise staging via sentinel lymph node biopsy (SLNB) to detect occult metastases early, significantly improving five-year survival [6], alongside prolonged dermatologic follow-up to monitor recurrence or late metastasis [6]. Ultra-late recurrences (>10 years post-diagnosis) occur in <5% of melanoma cases [7, 8], underscoring melanoma's unpredictable biology and justifying lifelong surveillance. Standard management involves wide local excision ± SLNB.

Revolutionarily, systemic therapies for advanced melanoma have evolved over the past decade through immunotherapy (anti-PD-1/CTLA-4 agents) and BRAF/MEK inhibitors for BRAF-mutated tumors, substantially improving outcomes in advanced disease [5,8]. This report details a 57-year-old Caucasian male patient with extensive metastatic amelanotic melanoma recurring 13 years post-diagnosis, highlighting long-term follow-up imperatives and modern diagnostic/therapeutic paradigms.

## Case presentation

The 57-year-old male patient presented to the emergency department with a chief complaint of nonspecific abdominal pain. His medical history was significant for a previous diagnosis of amelanotic melanoma located on the right foot, established in 2010, for which he had received appropriate surgical treatment but subsequently lost medical follow-up. The patient had not maintained regular dermatological surveillance after his initial diagnosis, which represents a critical factor in the evolution of his case. Two months before the current admission, the patient developed severe, pressure-like pain in the left upper quadrant, which was non-radiating and partially responsive to analgesia.

**Figure 1:**
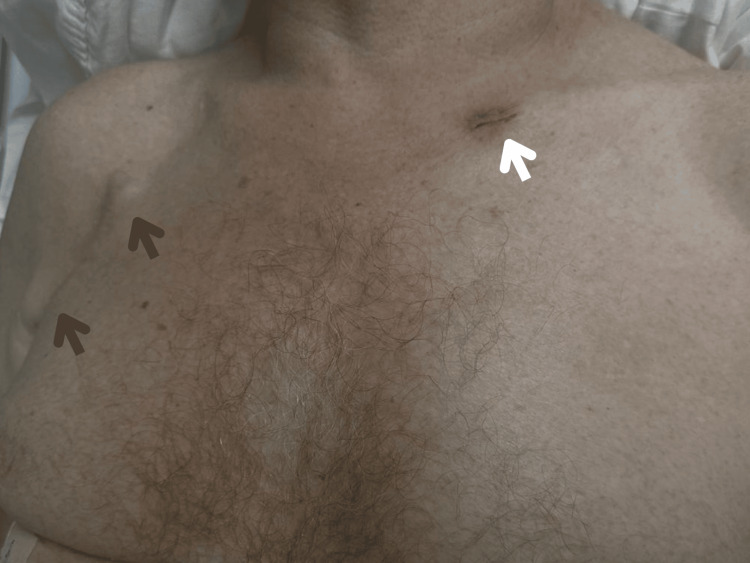
Site of previous cutaneous metastasis where the biopsy was taken (white arrow), and right axillary lymphadenopathy (dark arrows)

Physical examination findings included multiple 1 cm, reddish, smooth, firm, and asymptomatic cutaneous nodules on the thorax, lower extremities, and occipital area, along with bilateral submandibular and axillary lymphadenopathy (Figure 1).

**Figure 2:**
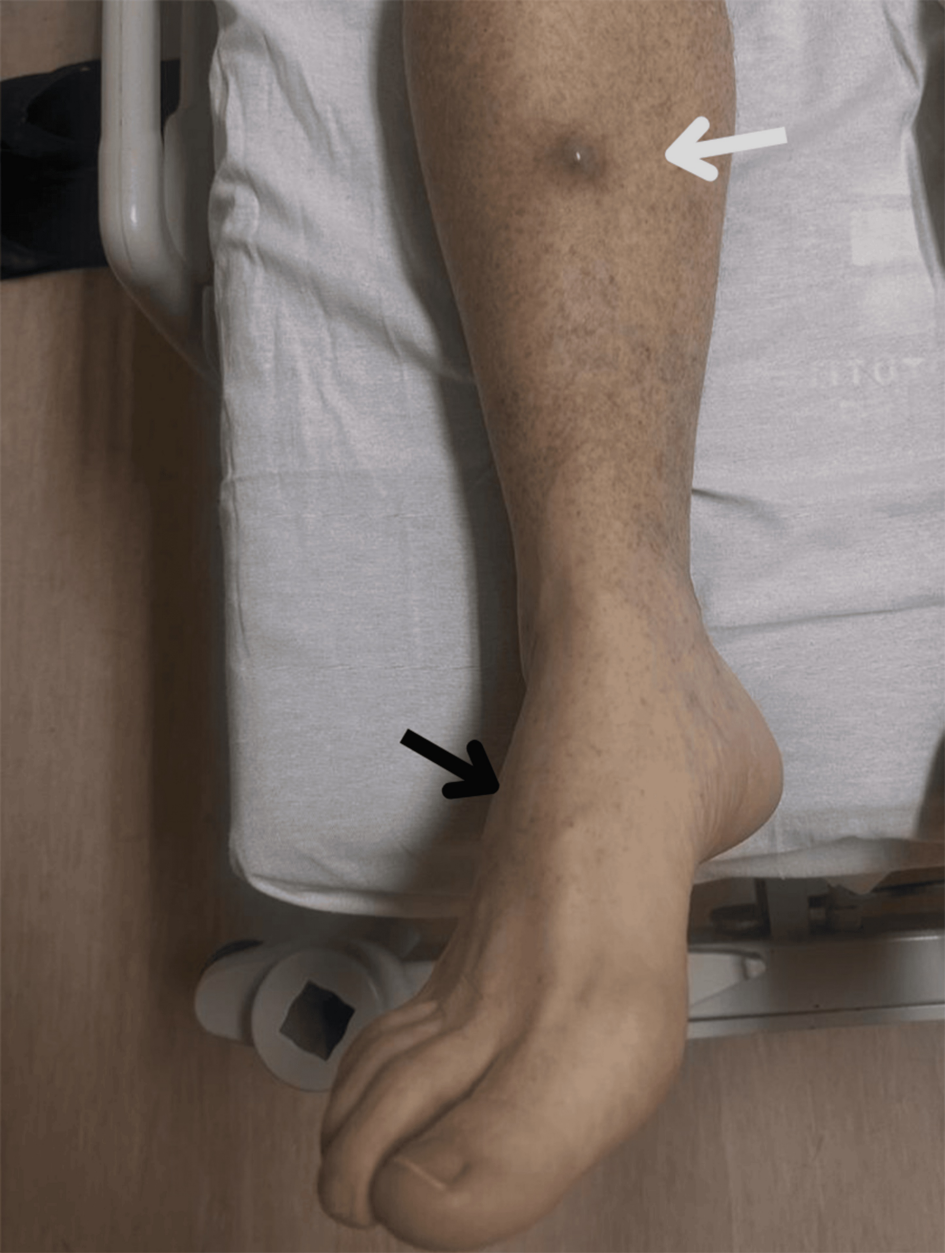
Location of the previous biopsy (white arrow), and nodular cutaneous metastasis (dark arrow) on the right lower extremity

**Figure 3:**
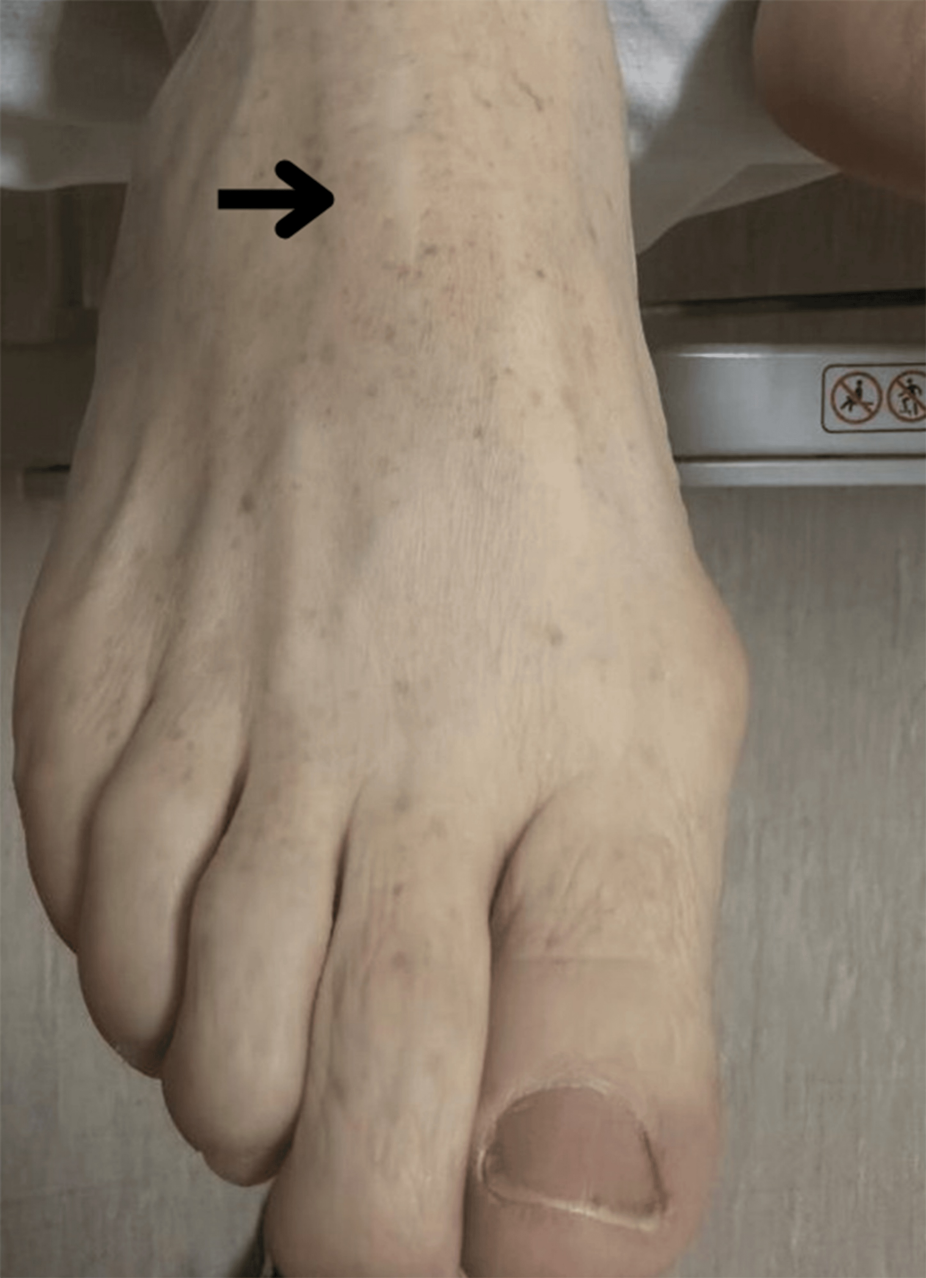
Previous biopsy site on the right forefoot (dark arrow)

Hepatosplenomegaly was also observed. A 3x2 cm mobile, non-tender nodular dermatosis on the lower right leg was highly suggestive of metastasis, given its proximity to the 3 cm scar from the initial foot biopsy (Figures 2, 3).

**Figure 4:**
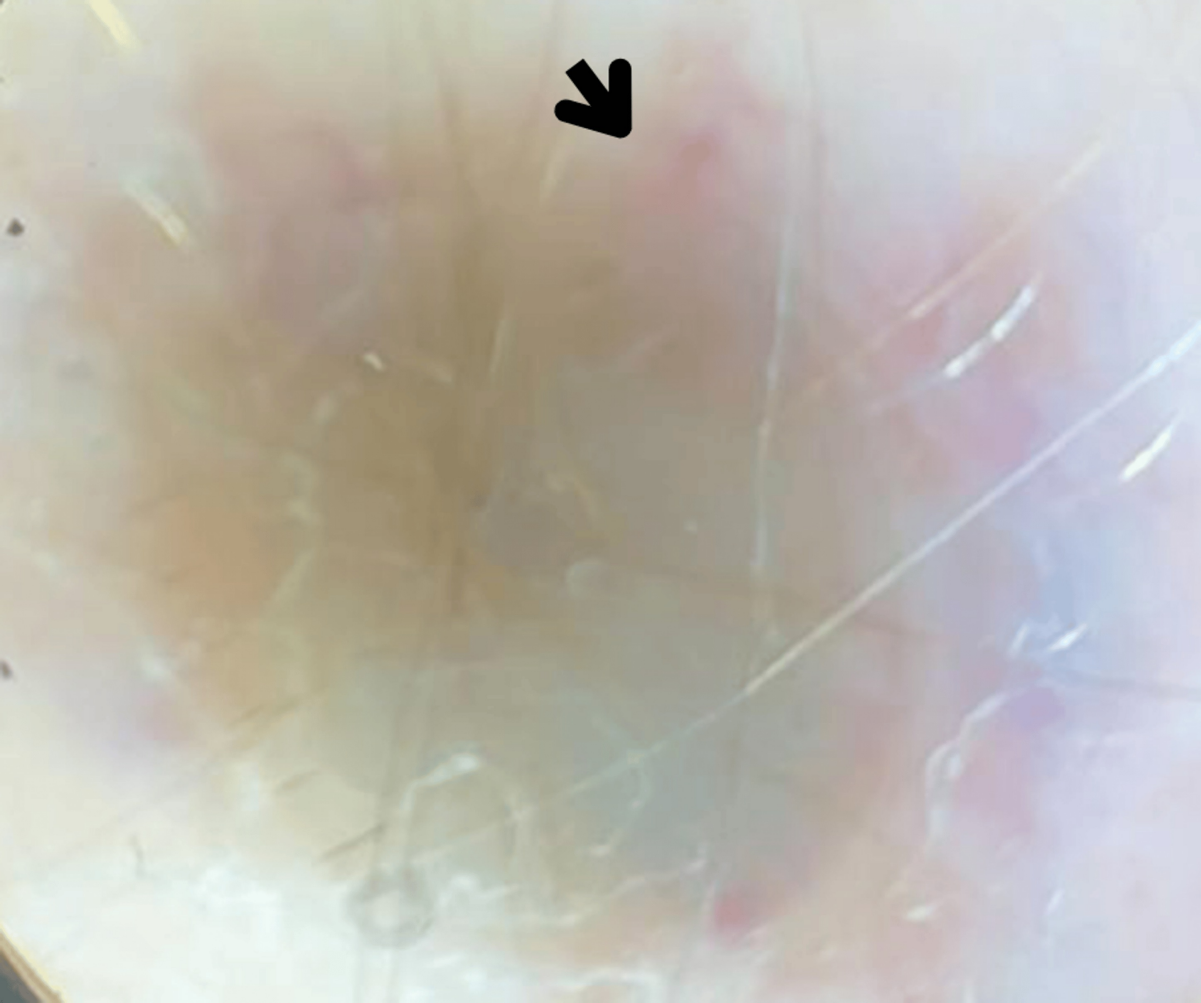
Dermoscopy revealed a nodule approximately 1 cm in diameter, characterized by pale pink or reddish structureless areas (dark arrow), resembling a "milky-red" pattern, and vascular polymorphism

Non-polarized dermatoscopy of an occipital cutaneous metastasis demonstrated pale pink, structureless "milky-red areas" and polymorphous atypical vascular patterns, including irregular linear, dotted, and corkscrew vessels, interspersed within the lesion (Figure 4). 

Initial laboratory values are detailed in Table 1. The patient's laboratory profile demonstrated hematologic and renal derangements, collectively indicating systemic compromise from advanced metastatic disease. This included severe microcytic anemia (hemoglobin {Hb} 7 g/dL, hematocrit {Hct} 23.9%, mean corpuscular volume {MCV} 83.9 fL), likely reflecting bone marrow infiltration, chronic inflammation, or occult blood loss from gastrointestinal metastases. Marked leukocytosis (WBC 17.8×103/μL) with absolute neutrophilia (14.13×103/μL) and reactive thrombocytosis (751×103/μL) suggested a leukemoid reaction driven by tumor necrosis, cytokine release, or early medullary response to metastatic colonization. Concurrent acute kidney injury (Cr 1.9 mg/dL, BUN 27.1 mg/dL, urea 58 mg/dL) may have arisen from dehydration, obstructive uropathy due to retroperitoneal lymphadenopathy, paraneoplastic glomerulopathy, or tumor lysis effects. This triad of anemia, neutrophilia-driven leukocytosis, and renal dysfunction underscored multisystem failure attributable to aggressive metastatic dissemination.

**Table 1:** Initial laboratory test

Lab test	Patient result	Normal range	Units
White blood cells (WBC)	17.8	4.0 - 11.0	x 10^3/µL
Hemoglobin (Hb)	7	13.5 - 17.5 (male)	g/dL
Hematocrit (Hct)	23.9	38.8 - 50.0 (male)	%
Mean corpuscular volume (MCV)	83.9	80 - 100	fL
Platelets	751	150 - 450	x 10^3/µL
Neutrophils (absolute)	14.13	1.8 - 7.7	x 10^3/µL
Lymphocytes (absolute)	1.23	1.0 - 4.8	x 10^3/µL
Creatinine (Cr)	1.9	0.7 - 1.3 (Male)	mg/dL
Urea	58	20 - 40	mg/dL
Blood urea nitrogen (BUN)	27.1	7 - 20	mg/dL

**Figure 5:**
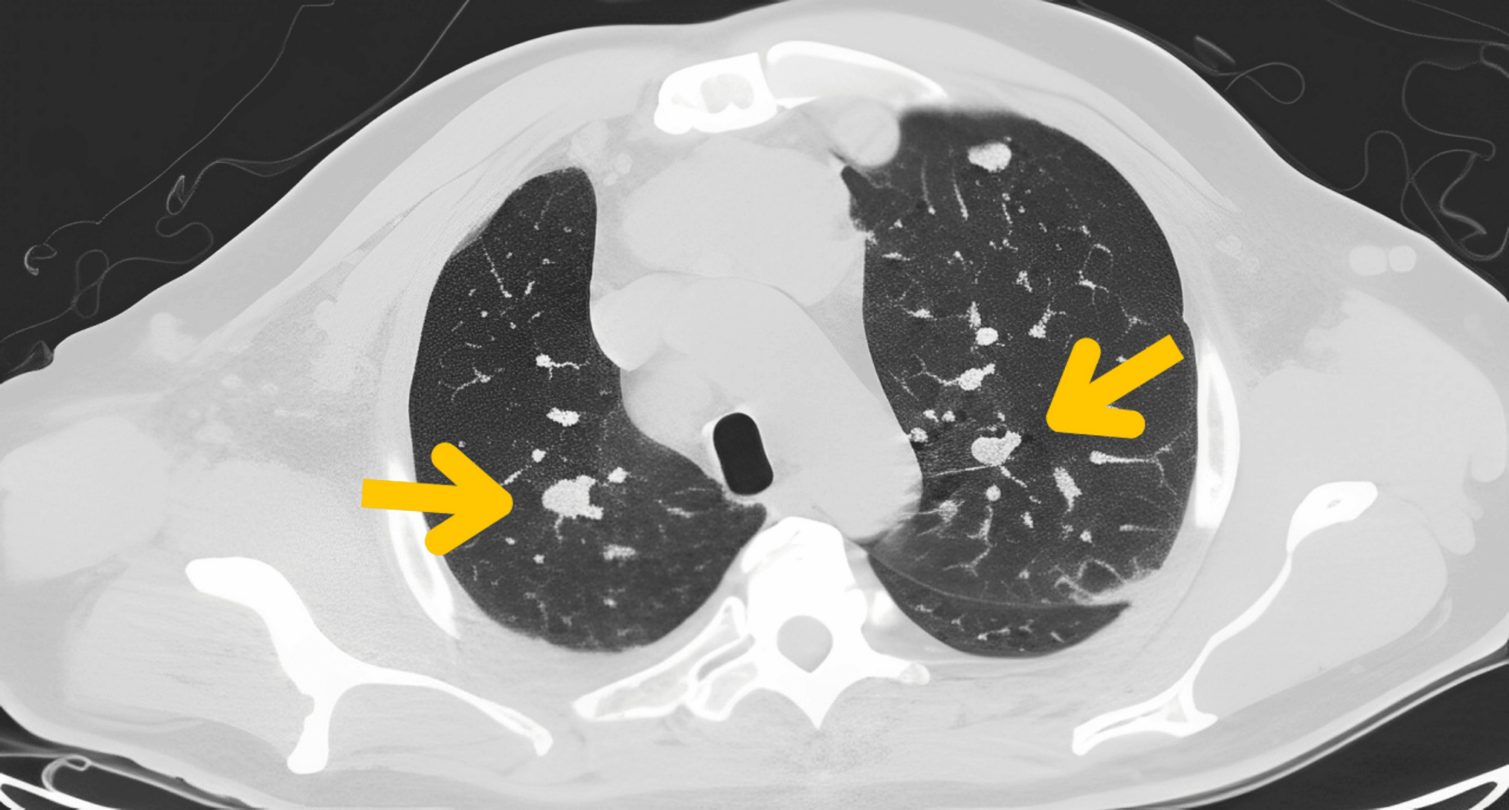
Axial non-contrast chest CT showing bilateral pulmonary nodules (yellow arrows) of heterogeneous size, a classic "cannonball" metastatic morphology

A computed tomography (CT) scan of the thorax, performed for the workup of these symptoms, revealed an extensive systemic disease burden. At the thoracic level, multiple pulmonary nodules of variable size and density were identified in both lung fields, consistent with pulmonary metastases (Figure 5).

**Figure 6:**
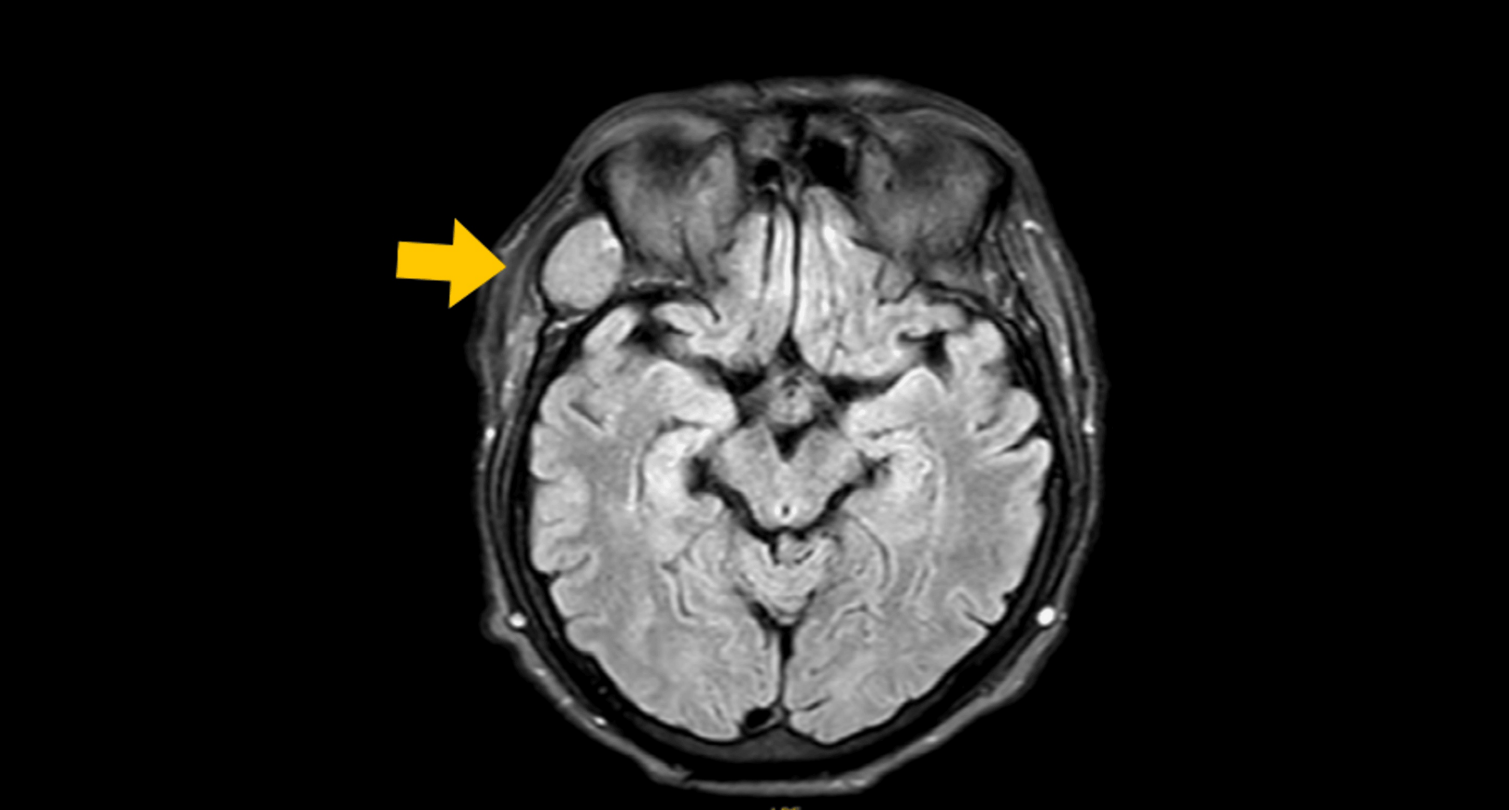
Axial FLAIR brain MRI showing a prominent hyperintense lesion (yellow arrow) in the right orbit/temporal region, the finding consistent with an orbital metastasis FLAIR: fluid-attenuated inversion recovery

**Figure 7:**
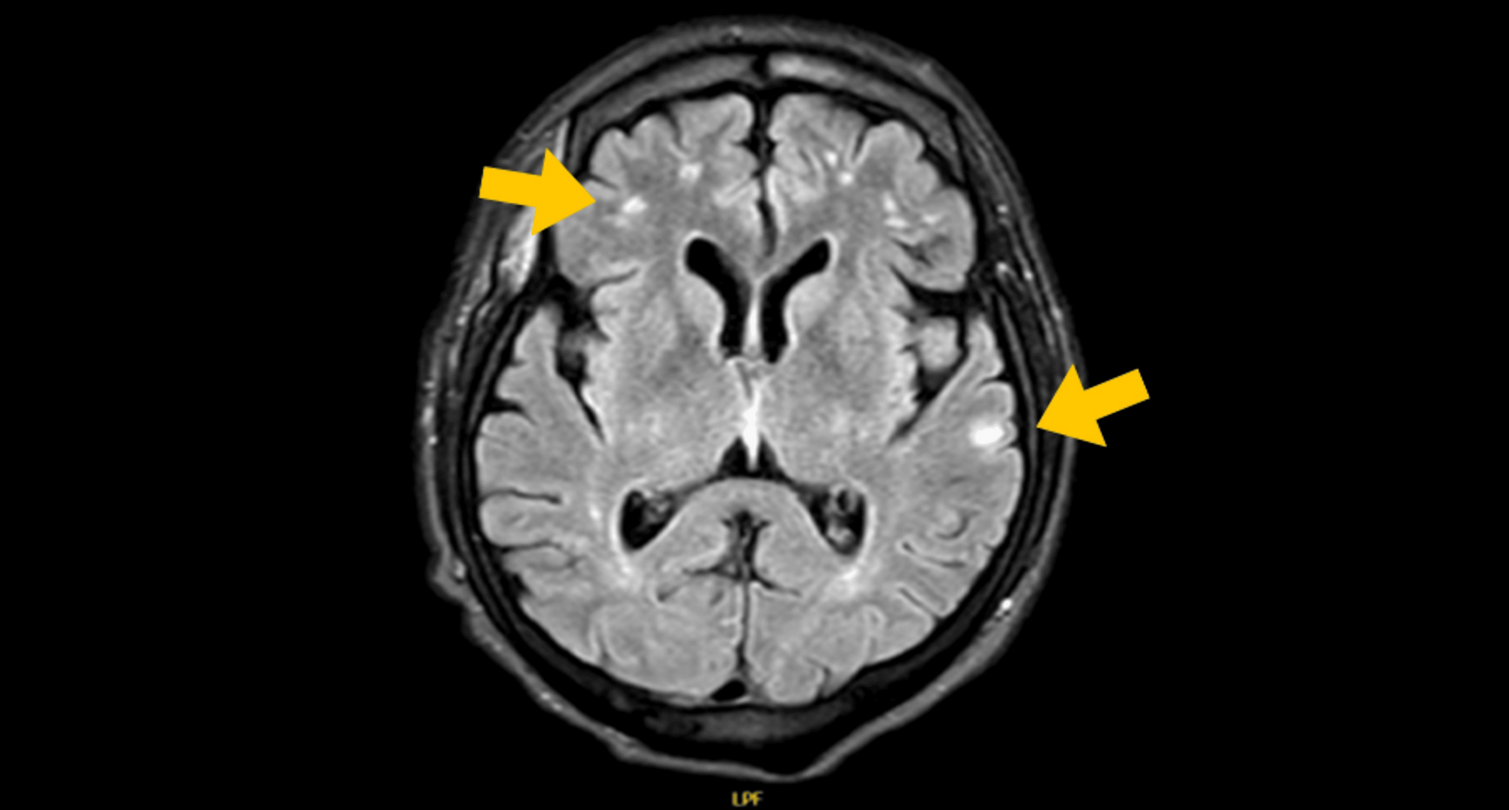
FLAIR MRI showing multiple hyperintense subcortical nodules (yellow arrows) with extensive vasogenic edema (right parietal lesion most prominent) FLAIR: fluid-attenuated inversion recovery

Given the extent of the disease, the workup was supplemented with a magnetic resonance imaging (MRI) of the brain. This study confirmed the presence of multiple intraparenchymal nodular lesions, which appeared predominantly hypointense or isointense on fluid-attenuated inversion recovery (FLAIR) sequences (Figures 6, 7), many surrounded by significant vasogenic edema-findings characteristic of cerebral metastases.

**Figure 8:**
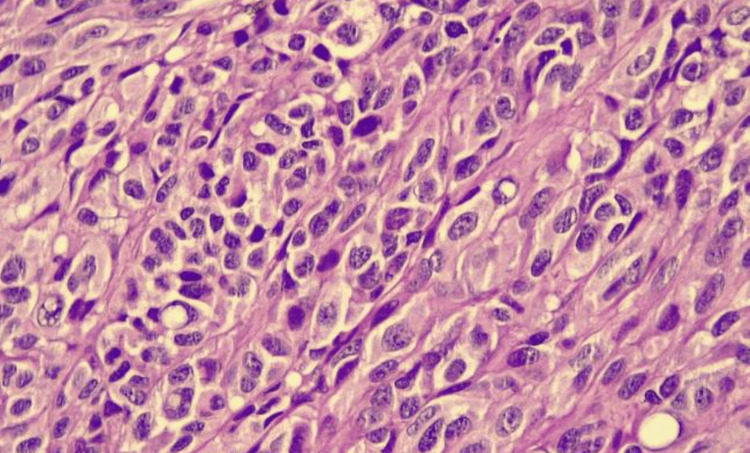
Hematoxylin and eosin stained histological section, the malignant neoplasm composed of regular nests The cells are medium to large in size, characterized by round nuclei, prominent nucleoli, eosinophilic cytoplasm, significant atypia, and high mitotic figures, with no evidence of pigment. A sparse lymphocytic inflammatory infiltrate, focal hemorrhage, and necrotic areas are also noted.

A diagnostic biopsy of a left anterior cervical lymph node yielded histopathological confirmation of metastatic epithelioid amelanotic melanoma (Figure 8). Immunohistochemical analysis supported the diagnosis of metastatic amelanotic melanoma (Table 2). The histopathological findings obtained unequivocally confirmed the diagnosis of metastatic amelanotic melanoma.

**Table 2:** Immunohistochemistry of cutaneous metastasis in the thorax

Antibody	Control	Result
Melan-A (Bio&SB)	Adequate	Positive in neoplastic cells
CDX10 (Bio&SB)	Adequate	Positive in neoplastic cells
S100 (Bio&SB)	Adequate	Positive in neoplastic cells
Cytokeratin AE1/AE3	Adequate	Negative

Subsequently, the patient was referred to the oncology service for consideration of appropriate systemic therapy. Given the extent of documented metastatic disease, therapeutic options considered included immunotherapy using anti-PD-1 agents such as nivolumab or pembrolizumab, or alternatively, combination therapy using ipilimumab plus nivolumab, depending on the patient's functional status and specific molecular characteristics of the tumor.

## Discussion

Amelanotic melanoma constitutes a clinically complex variant of cutaneous melanoma characterized by distinctive epidemiological and morphological features that present unique diagnostic and management challenges in contemporary practice [1,2]. This complexity stems from several interconnected factors that fundamentally distinguish it from conventional pigmented melanomas.

Epidemiologically, amelanotic melanoma demonstrates a distinct predilection for older Caucasian patients, particularly those over 50 years of age with Fitzpatrick skin types I-II, who frequently present with asymptomatic nodular lesions that lack the classical warning signs of pigmented melanomas [1,2]. The absence of pigmentation represents the most significant morphological characteristic, as it eliminates the primary visual cue that typically alerts both patients and clinicians to potential malignancy. This depigmented appearance results from deficient melanin production or complete absence of melanocytes capable of melanin synthesis, creating lesions that appear as flesh-colored, pink, or red nodules rather than the expected dark pigmented growths [1,2].

This case exemplifies established epidemiological patterns through a Caucasian male patient over 50 years of age presenting with asymptomatic nodular lesions, consistent with reports that 58% of amelanotic melanomas manifest as papules or nodules [4]. The 13-year interval to metastatic recurrence represents an ultra-late recurrence, a phenomenon documented in less than 5% of melanoma cases [6,7], highlighting this malignancy's unpredictable biology and underscoring the imperative for lifelong surveillance extending beyond conventional follow-up periods.

Diagnostic difficulties persist due to the lesion's depigmented appearance and clinical similarity to benign entities such as pyogenic granuloma, Spitz nevus, and seborrheic keratosis, alongside malignancies including basal cell carcinoma and cutaneous lymphoma [4,8]. Dermatoscopic evaluation proved indispensable in this case, revealing pathognomonic milky-red areas (70% specificity) and atypical vascular patterns exhibiting 89% sensitivity [4,9], including irregular linear, punctate, and corkscrew vessels with asymmetric distribution. These findings significantly elevated clinical suspicion and guided diagnostic confirmation. Advanced imaging modalities were equally critical, with chest computed tomography identifying "cannonball" pulmonary metastases [10] and cerebral magnetic resonance imaging demonstrating 90% sensitivity for brain metastases larger than 3 mm [11], enabling comprehensive disease staging.

Immunohistochemical profiling (S100⁺, Melan-A⁺, CDX10⁺) confirmed melanocytic origin, with S100 showing 95% sensitivity and Melan-A providing enhanced specificity [12]. The molecular heterogeneity inherent to amelanotic melanoma, particularly variable BRAF mutation status, carries significant therapeutic implications for targeted therapy selection [13,14]. The contemporary therapeutic landscape has undergone transformative evolution, where anti-PD-1 monotherapy (nivolumab/pembrolizumab) achieves 40-60% objective response rates and two to three year overall survival [15], while ipilimumab-nivolumab combination therapy yields five-year survival in 50% of cases [15]. For BRAF-mutant tumors, BRAF/MEK inhibitors provide one-and-a-half to two years progression-free survival [15,16], representing a >100% improvement over historical chemotherapy outcomes [17,18].

This case underscores four critical imperatives: sustained diagnostic vigilance for depigmented lesions in high-risk populations; multidisciplinary integration of dermatoscopy, advanced imaging, and molecular profiling; guaranteed access to modern systemic therapies; and implementation of lifelong surveillance protocols transcending traditional five-year frameworks. The patient's loss to follow-up precluded adjuvant therapy, exposing systemic challenges in maintaining longitudinal care. Future research should prioritize biomarkers for early recurrence detection, risk-stratified surveillance protocols, and optimized diagnostic tools.

## Conclusions

This case paradigmatically exemplifies the multifaceted challenges of amelanotic melanoma, fundamentally encompassing delayed diagnosis due to depigmented presentation and ultra-late metastatic recurrence. These findings underscore the critical importance of implementing comprehensive strategies in contemporary clinical practice. Enhanced diagnostic awareness requires healthcare professionals to maintain heightened clinical suspicion for depigmented lesions, particularly among populations with clearly identified elevated risk factors. Advanced diagnostic tools, specifically dermatoscopy and cross-sectional imaging, are essential for achieving precise diagnoses and accurate disease staging.

Extended surveillance protocols remain indispensable, as lifelong follow-up is necessary given the well-documented potential for ultra-late recurrences inherent to this neoplasm. Guaranteed access to modern therapies, including contemporary immunotherapy and targeted agents, significantly improves clinical outcomes even in advanced metastatic disease. The effective integration of these core elements into daily clinical practice is crucial for optimizing patient outcomes and substantially reducing morbidity and mortality associated with this particularly aggressive malignancy.
